# What is the impact of interventions that prevent fetal mortality on the increase of preterm live births in the State of Sao Paulo, Brazil?

**DOI:** 10.1186/s12884-015-0572-6

**Published:** 2015-07-23

**Authors:** Gizelton Pereira Alencar, Zilda Pereira da Silva, Patrícia Carla Santos, Priscila Ribeiro Raspantini, Barbara Laisa Alves Moura, Marcia Furquim de Almeida, Felipe Parra do Nascimento, Laura C Rodrigues

**Affiliations:** Department of Epidemiology, University of São Paulo, School of Public Health, São Paulo, Brazil; Department of Preventive Health, University of São Paulo, School of Medicine, São Paulo, Brazil; Faculty of Epidemiology and Population Health, London School of Hygiene and Tropical Medicine, London, UK

**Keywords:** Fetal mortality, Perinatal mortality, Preterm births, Cesarean section, Gestational age, Prenatal care, Multiple births, Maternal schooling, Time trend

## Abstract

**Background:**

There is a global growing trend of preterm births and a decline trend of fetal deaths. Is there an impact of the decline of fetal mortality on the increase of preterm live births in State of Sao Paulo, Brazil?

**Methods:**

The time trends were evaluated by gestational age through exponential regression analysis. Data analyzed included the fetal mortality ratio, proportion of preterm live births, fertility rate of women 35 years and over, prenatal care, mother's education, multiple births and cesarean section deliveries. A survival analysis was carried out for 2000 and 2010.

**Results:**

Preterm births showed the highest annual increase (3.2 %) in the less than 28 weeks of gestation group and fetal mortality ratio decreased (7.4 %) in the same gestational age group. There was an increase of cesarean section births and it was higher in the < 28 weeks group (6.1 %). There was a decreased annual trend of mothers with inadequate prenatal care (6.1 %) and low education (8.8 %) and an increased trend in multiple births and fertility rates of women of 35 years and over. The variables were highly correlated to which other over time. In 2000, 8.2 % of all pregnancies resulted in preterm births (0.9 % in fetal deaths and 7.3 % in live births). In 2010, the preterm birth increased to 9.4 % (0.8 % were preterm fetal deaths and 8.6 % preterm live births).

**Conclusions:**

The results suggest that 45.2 % could be the maximum contribution of successful interventions to prevent a fetal death on the increase in preterm live births. This increasing trend is also related to changes of the women reproductive profile with the change of the women reproductive profile and access to prenatal care.

## Background

The proportion of preterm births is growing in several countries [1]. The global incidence of preterm births is estimated at 9.6 % in 2005. The highest rates were found in the USA (10.6 %) and some African countries (11.9 %), whereas the lowest rates were observed in Europe (6.2 %). In Brazil, the trend towards preterm births is the same as the global trend [2].

The reduction of neonatal and infant mortality in Brazil is well studied [3, 4], whereas little is known about trends and patterns in fetal mortality. Previous analysis of primary data showed that 90 % of fetal deaths are antepartum in large Brazilian cities, like São Paulo [5] and Rio de Janeiro [6].

Preterm births can result from three situations: medical intervention (induced vaginal birth or elective cesarean section), premature rupture of membranes and spontaneous premature labor, which correspond to roughly 25 %, 25 % and 50 % of the preterm births respectively, in countries such as Canada and USA [7]. A multicentre study in 20 tertiary hospitals in Brazil found that of the preterm births were 35.4 % due to therapeutic interventions, 35.9 % due to spontaneous onset and 28.7 % due to premature rupture of membranes [8].

In these countries, the increase in preterm births was accompanied by a decrease in perinatal mortality [8–10], especially among pregnancies of 34 and 36 weeks of length of gestation [11]. This is consistent with the hypothesis that early delivery, in high risk pregnancies, can increase the survival rate of fetuses and therefore reduce perinatal mortality [12, 13]. This hypothesis is supported by the similarity between risk factors for preterm births and fetal and neonatal deaths [12]. These include some maternal characteristics: age, level of schooling, socioeconomic level, nutritional status [14, 15], presence of morbidities [16] and ethnic group [17, 18]; and some factors associated with the pregnancy: previous premature birth [10, 19], multiple births, assisted reproduction [20], biological markers [19], vaginal inflammatory infections [21], hemorrhage, uterine hypertrophy or cervical incompetence [22]. Several Brazilian studies have shown that inadequate prenatal care is also found among the risk factors for preterm births [23, 24], fetal and neonatal deaths [5, 6].

Brazil does not have population-based information that enables to identify the proportion of preterm births due to medical indication, premature rupture of membranes or spontaneous premature labor and the contribution of each sub-group to the observed increase in preterm births. Additionally, it is not possible to identify whether there was a shift to the left side of the curve of distribution of births by gestational age, because this variable is registered in groups of gestational age at the live birth certificates until 2011 [25]. However, a multicentre study in 20 tertiary hospitals in Brazil found that of the preterm births were 35.4 % due to therapeutic interventions, 35.9 % due to spontaneous onset and 28.7 % due to premature rupture of membranes [26].

However, the State of São Paulo has high-quality information [27] available, which shows a reduction in neonatal and fetal mortality and an increase in preterm births [28]. The present study aimed to assess the time trend of preterm live births and fetal deaths according to gestational age groups and certain indicators associated with the profile of pregnant women, to contribute to a better understanding of possible relationships between the trends.

## Methods

An ecological study of historical series was conducted, assessing data on live births, fetal deaths and cesarean sections (CS) from 2000 to 2010, from mothers living in the State of São Paulo, Southeastern Brazil.

The following databases managed by the Ministry of Health of Brazil were used: Mortality and Live Birth Information Systems [29]. The fertility rate of women 35 years and over was obtained from health indicators of the Health Information Inter-Agency Network [28].

Live births (LB), fetal deaths and proportion of live births by CS were analyzed according to gestational age, divided into three categories: <28; 28–31; 32–36, and for CS we included 37–41 weeks. The proportion of preterm live births (<37 weeks), the fetal mortality ratio (fetal deaths/LB x 1,000) and neonatal mortality rate (neonatal deaths/LB x 1,000) were calculated. We used the fetal mortality ratio, rather than fetal mortality rate, to have the same denominator for both measures.

As an indicator of inadequate prenatal care, the proportion of live births in pregnancies with fewer than four prenatal care appointments was used. The proportion of live births from mothers with fewer than eight years of schooling was considered an indicator of low maternal level of education. Additionally, the proportion of live births from multiple pregnancies and the fertility rate of women aged 35 years and over (live births of women 35 years and over/women population 35 years and over) were used in this study.

Exponential regression was used to assess time trends for all variables studied. Pearson correlation coefficients were calculated to evaluate the association between the time trends of the variables studied.

A proxy for the total number of pregnancies was obtained from the sum of live births and fetal deaths for 2000 and 2010. We treated all the pregnancies as at risk of two outcomes: fetal death and preterm births [30]. This was considered as a gestation cohort, where the denominator the total number of gestations at each gestational age, and a Kaplan-Meier survival graph was used. The estimate of survival rate according to gestational age group was calculated and the proportions of preterm fetal deaths and preterm live births pregnancies were obtained.

We calculated the maximum increase in preterm births that could have resulted from a successful intervention preventing a fetal death and resulting in a preterm birth during the period, by calculating the overall reduction in fetal deaths and increase in preterm births over the period and presenting the reduction in fetal deaths as a proportion of the increase in preterm births.

The data employed in this study is public and it is freely available through the site of Brazilian Health Ministry (www.datasus.gov.br). This study has no conflict of interests and the research project was approved by the Research Ethics Committee of the School of Public Health of the University of Sao Paulo.

## Results

The proportion of incomplete data on gestational age (GA) was lower than 3 % for live births and approximately 10 % for fetal deaths; this was less than 5 % for all other variables analyzed.

**Fig. 1:**
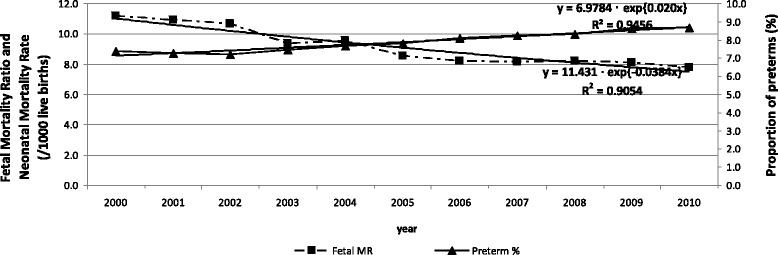
Fetal mortality ratio and proportion of preterm live births. State of São Paulo, 2000–2010

Figure 1 shows an average increase in the number of preterm births of 2.0 % per year, rising from 7.4 % in 2000 to 8.7 % in 2010. The average reduction in the fetal mortality ratio was 3.9 % per year, decreasing from 11.3 to 7.8 fetal deaths per 1,000 LB, and in the neonatal mortality rate this was 3.3 %, from 12.1 to 8.1 deaths per 1,000 LB. All these time trends were statistically significant.

**Fig. 2:**
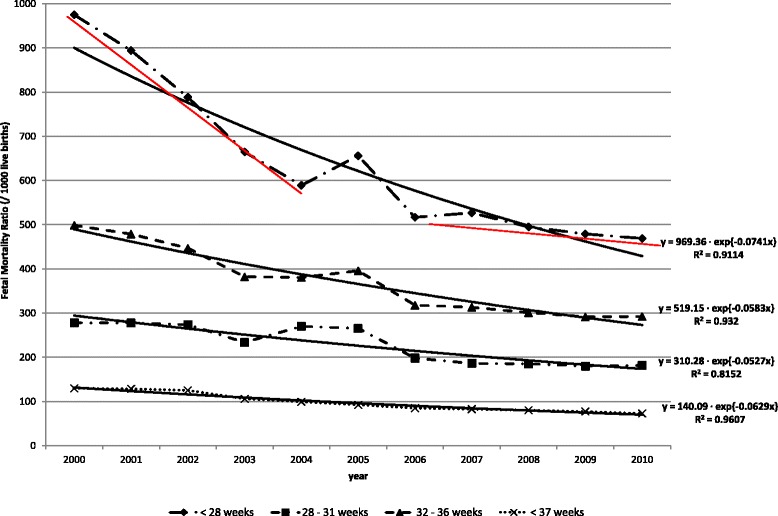
Fetal mortality ratio by gestational age. State of São Paulo, 2000–2010

The total number of fetal deaths was 7,535 in 2000 and 4,688 in 2010. Fetal deaths were predominantly preterm (more than 80 %), of which 5.9 % at less than 28 weeks; 9.5 %, between 28 and 31 weeks; and 85.6 %, between 32 and 36 weeks, in 2010. The fetal mortality ratio decreased with the increase in gestational age (Fig. 2). The reduction in fetal mortality ratio was higher among preterm pregnancies (6.2 % per year). The group of fetal deaths with a gestational age less than 28 weeks had a greater reduction through time (7.4 % per year), followed by the group of 32–36 weeks (5.8 % per year) and, lastly, the group of 28–31 weeks (5.3 % per year), all of which were statistically significant (Fig. 2). The mortality decrease among term pregnancies (37 weeks and over gestation duration) was lower than at others gestational age groups, and it was not statistically significant (y = 1.9535e^-0.019x^; R^2^ = 0.31; p = 0.08). The reduction in fetal mortality of post-term pregnancies (42 weeks and over) in this period was not statistically significant (y = 34.016e^-0.078x^; R^2^ = 0.28; p = 0.09) (data not shown). There was a small number of deaths in this group: 148 in 2000 and 25 in 2010.

**Fig. 3:**
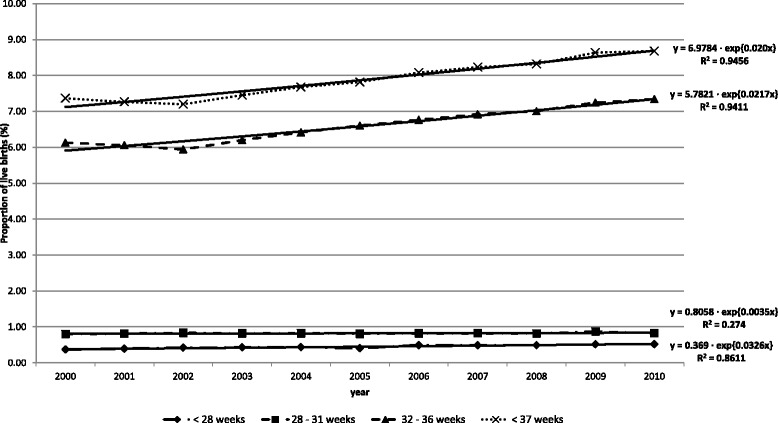
Proportion of preterm live births by gestational age. State of São Paulo, 2000–2010

The total number of preterm live births was 48,581 in 2000 and 52,129 in 2010. The distribution of gestational age in preterm live births in 2010 was: *<*28 weeks, 5.6 %; 28–31 weeks, 9.4 %; and 32–36 weeks, 84.9 %. The proportion of preterm births increased in all gestational ages: the highest annual growth rate occurred in the group of extreme premature births (<28 weeks): 3.3 %; and very premature (28–31 weeks): 0.4 %; and the proportion of moderate premature births (32–36 weeks) increased 2.2 % per year (Fig. 3).

**Fig. 4:**
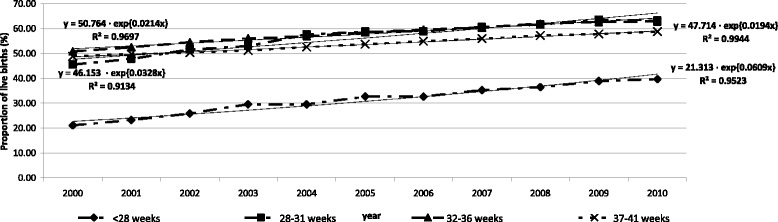
Proportion of live birth delivery by cesarean section by gestational age. State of São Paulo, 2000–2010

There was an increase in the proportion of live births by CSs (Fig. 4). The distribution of proportion of live births by CS according to gestational age, in 2010 was: *<*28 weeks, 39.6 %; 28–31 weeks, 63.2 %; and 32–36 weeks, 62.8 % and 37 weeks and over: 58.7 %. The proportion of births by CS increased in all gestational ages but it was highest in the lowest gestational ages: in extreme premature births (<28 weeks): 6.1 %; very premature (28–31 weeks): 3.3 %, moderate premature births (32–36 weeks), 2.1 %. The annual average increase in CS in term births was 1.9 %.

The profile of pregnant women in the State of São Paulo changed, during this period (Table 1). There was an average reduction of 8.8 % per year in the proportion of mothers with fewer than eight years of schooling (y = 54.2e^-0.088x^; R^2^ = 0.99; p < 0.001), which was statistically significant. Meanwhile, there was also an improvement in access to prenatal care, with a statistically significant reduction (y = 8.15e^-0.061x^; R^2^ = 0.88; p < 0.001) in the proportion of mothers with inadequate prenatal care (less than four appointments) There was an increase in the proportion of live multiple births (y = 1.86e^0.0193x^; R^2^ = 0.90; p < 0.001). During this period, there was a slight increase in the fertility rate of women aged 35 years and more (Table 1). This group of women accounted for 9.4 % of births in 2000 and 13.0 % in 2010. The proportion of primiparous women aged more than 35 years was 15.1 % in 2000 and 21.4 % in 2010 (data not shown).

**Table 1:** Characteristics of live births and fertility rate of women aged 35 years and more. State of São Paulo. 2000–2010

Year	% mothers less than 8 years of study	% less than 4 prenatal care appointments	% multiple gestation	Fertility rate of women of 35 years and over^a^
2000	48.58	8.25	1.88	0.11
2001	45.20	7.75	1.91	0.11
2002	41.91	7.07	2.03	0.12
2003	38.68	6.02	2.00	0.12
2004	35.43	5.52	2.02	0.13
2005	32.63	5.18	2.17	0.13
2006	29.51	5.08	2.16	0.13
2007	26.99	4.83	2.13	0.14
2008	24.33	4.50	2.21	0.14
2009	22.36	4.80	2.32	0.15
2010	20.62	4.64	2.25	0.15

^a^live births of women 35 years and over/women population 35 years and over

Source: Ministry of Health of Brazil

Table 2 shows the correlation matrix among the time trends in variables studied. Trends in variables were correlated to each other and to the trends in preterm births. There was a negative correlation between trends in preterm births and in fetal mortality ratio as the proportion of preterm increased, and of fetal deaths decreased over the period. There was a positive correlation between the trend in proportion of multiple births and in preterm births: both increased over the time period. There were negative correlations between trend in preterm births (which increased over the time period) and the trends in the proportion of women with few years of schooling and with inadequate prenatal care, both of which decreased over the period. There was a positive correlation between the trend in fertility rate of 35 years and over women and in preterm births (both increased over the time period). There was a negative correlation between trends in inadequate prenatal care (which decreased over time) and in the proportion of multiple births (which increased over time). There was a negative correlation between the trend in multiple births (which increased over time) and the trend in few years of schooling (which decreased over time). Finally, there was a negative correlation between the trend in proportion of mothers with a few years of schooling (which decreased over time) and the trend in fertility rate of women aged 35 years and more which increases over time.

**Table 2:** Pearson correlation matrix among trends of preterm live birth characteristics, fertility rate and fetal mortality ratio. State of São Paulo. 2000–2010

Variables	Preterm live birth less than 36 weeks	% mothers lest than 4 prenatal appointments	% multiple gestation	% mothers less than 8 years schooling	Fertility rate of women of 35 years and over	Fetal mortality ratio
% mothers less than 4 prenatal appointments	−0.866					
% multiple gestation	0.915	−0.886				
% mothers less than 8 years schooling	−0.969	0.949	−0.950			
fertility rate of women of 35 years and over	0.967	−0.925	0.948	−0.995		
fetal mortality ratio	−0.953	0.969	−0.910	0.979	0.960	
% live birth by cesarean	0.984	−0.922	0.941	−0.996	0.993	−0.971

p < 0.001 for all correlation coefficients

Source: Ministry of Health of Brazil

**Fig. 5:**
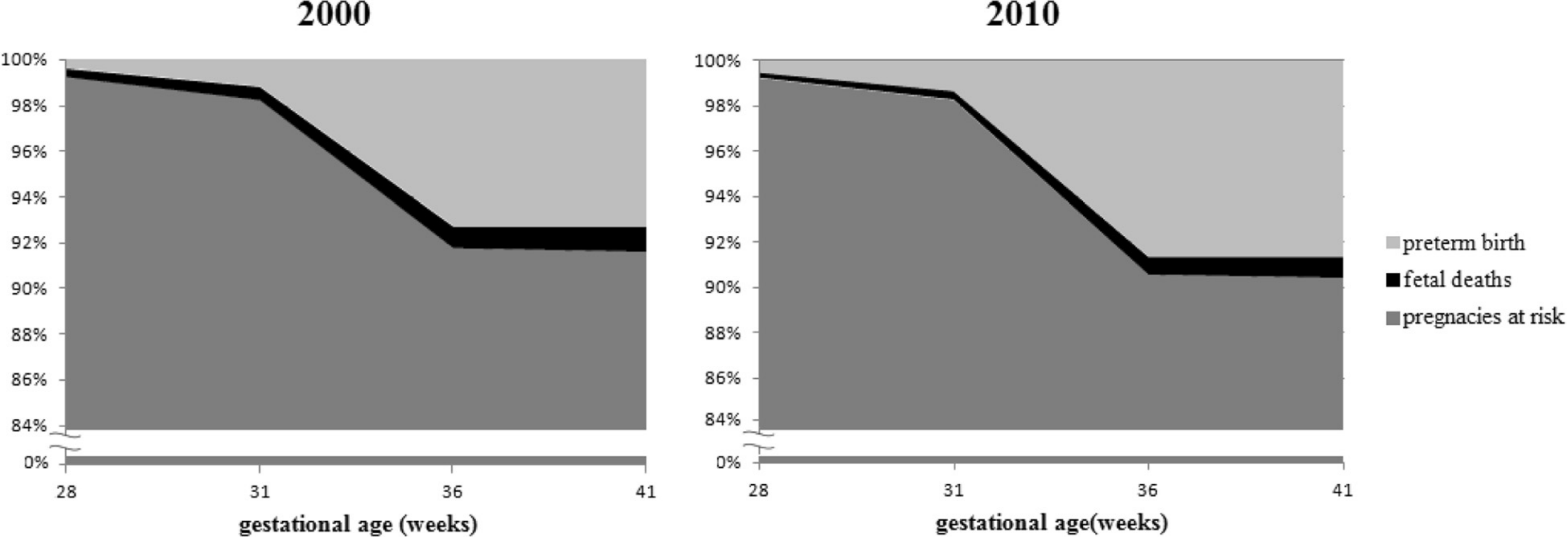
Distribution of pregnancies according to fetal deaths, preterm live birth and pregnancies at risk. State of São Paulo, 2000 and 2010

Figure 5 presents the data for pregnancies for each of the two time periods as a cohort of pregnancies followed up from the 28^th^ to the 41^st^ week. Pregnancies in the cohort can have negative two outcomes: preterm births (shown in light gray) and fetal deaths (shown in black). At each gestational age, pregnancies that did not end in fetal death or premature birth remain at risk and are shown in dark gray. All pregnancies that last until week 40 are term or post term births.

In 2000, 8.2 % of all pregnancies resulted in preterm births: 0.9 % in fetal deaths and 7.3 % in live births. In 2010, the total number of all pregnancies whose outcome was preterm birth increased to 9.4 %, of which 0.8 % corresponded to preterm fetal deaths and 8.6 %, preterm live births (Fig. 5).

During the period studied, there was a reduction of 1,604 fetal deaths, and an increase of 3,548 preterm live births. If all the reduction in fetal deaths were the result of a successful intervention leading to a live preterm birth, this would correspond to an additional 1604 preterm births. This would explain 45.2 % of the increase in preterm births over this period. This value represents the maximum contribution of successful interventions to prevent a fetal death on the increase in preterm live births, and it is likely that the contribution is less than this maximum value.

## Discussion

Our results show that the decline in fetal mortality, the increase in preterm births and the increase in proportion of cesarean section (CS) were all more marked in low gestational ages during the study period; these findings are consistent with an increase in adequate clinical interventions in early pregnancy preventing fetal deaths and contributing to the increase in preterm births. This could only explain a maximum of 45 % of the increase in preterm births.

The rates of fetal mortality in the State of São Paulo were lower than for Brazil and slightly higher than that in high income countries. The distribution of fetal deaths by gestational age is similar (although a little higher) to that in high income countries [31].

The proportion of preterm live births in the State of São Paulo (8.7 %) in 2010 was lower than that estimated for Brazil (9.2 %) [32] and lower than that found in a multicentre study based on tertiary hospitals in Southeast Brazil (11.1 %) [8]. It was similar to that estimated for Latin America (8.4 %) and some developed countries (8.6 %) but lower than in other high and middle income countries such as the USA (12.0 %) and India (15 %) [32]. The distribution of preterm births by gestational age was similar to that found internationally data [32], with a slightly lower contribution of moderate preterm births (32–36 weeks of gestation length). This small difference may result from the fact that not all countries follow the ICD-10 definition, which requires that all births are registered independent of the gestational age. Brazil follows the ICD-10 requirement [33]. In some countries, births less than 24 weeks of pregnancy are not recorded as live births [34, 35]. Another possible reason for this difference may be variations in the methods used to define gestational age (ultrasound, date of last menses or clinical measures) [34]. The method used to define the gestational age varies in Brazil and is not noted in the Brazilian live births certificates. However, the similarity between the results obtained and international data reassures of the robustness of the data and their usefulness.

The increase in the proportion of preterm births in the last decade in the State of Sao Paulo of approximately 2 % per year was higher than that found in Latin American countries (0.5 %) and developed countries (1.1 %) [33]. Changes in obstetric practices and the advance in intensive neonatal care in the last decades in industrialized and middle income countries may have contributed to the reduction in antepartum fetal deaths, as these are interrupted by performing cesarean sections or vaginal delivery induction, leading to preterm births [11].

There is some evidence that the growth in the number of preterm births is partly due to the obstetric interventions that are clinically recommended [8, 34, 36, 37]. Brazil and State of Sao Paulo have the world highest rates of CSs. The trend in increase in CSs was associated with the decline in preterm fetal mortality and with the increase of preterm births. The high numbers of CSs clearly indicate that some are unnecessary - but how many are necessary? A Brazilian study has shown that although about half the births by CS were preterm, only 17 % were of scheduled CS (which will include those done for reasons other than clinical indications) [37]. This is consistent with CS in low gestational ages (leading to preterm births) resulting from a clinical indication. In a study on Australia, around 91 % of therapeutic preterm <28 weeks births were from CS [38]. In our study, the fact that highest increase in preterm births, CS, and decrease in fetal mortality all happened in the extreme preterm births (<28 weeks) is again consistent with CSs in this gestational age group contributing to the survival of the fetuses.

These results are consistent with the hypotheses that obstetric intervention contributed to the decrease of fetal deaths and the increase of preterm births. No routine available population data identifies the contribution of medical indication to preterm births in Brazil. A multicentre study in tertiary hospitals found that 35 % of preterm births were therapeutic [26]. In our study, we described secular improvements in the proportion of women/deliveries with characteristics known to be risk factors for fetal death and for preterm births: low maternal schooling, proportion of pregnancies with inadequate prenatal care, multiple pregnancies and fertility of women over 35. Some improved over the time period: maternal schooling, proportion of pregnancies with adequate prenatal care, and others became more common: multiple pregnancies and fertility of women over 35.

Although babies born from women at younger ages (<20 years) is associated with preterm births [26, 37] in Brazilian studies using individual data, advanced maternal age (35 years and over) is also a risk factor to preterm live births [38–40]. Brazil experienced a demographic transition with a decline of adolescent fertility rate and an increase of the fertility rate of women of 35 years and over [28]. For this reason, in this study, we considered only trends in fertility of 35+ year old women; in addition, only births to older women, not younger women, have an increased risk of fetal mortality [41–44].

The increase of fertility rate of women aged 35 years and over and the increase in the proportion of primiparous women in this age group indicate that the women are postponing the beginning of their reproductive life, in addition to the growth of maternal education [45], like industrialized countries [41]. This change in reproductive behavior was also associated with the increase of multiple births, which showed a higher risk of occurrence of preterm live births and fetal deaths. Additionally, the increase of multiple births could also indicate positive changes in the quality of obstetric care available and its possible effect on the reduction of fetal mortality and increase of medically indicated preterm births.

The increase in years of maternal schooling (expressed in the table as a decrease in the proportion with low schooling) was associated with the decrease of fetal mortality and with the increase of preterm births. The increase of preterm births with women literacy was also found in an ecological study of preterm births from different countries [32] and the authors assumed that was an indication of western lifestyle leading to the increase of preterm births. The association between increase of literacy and decrease of fetal deaths and increase of preterm live births may be a result of the improved accesses to prenatal care. The improvement in access to prenatal care, suggests that the expansion in access may be enabling the identification of pregnancies of fetuses at a high risk of antepartum death [36, 43] and the interruption of a pregnancy can increase their chance of survival, as there is neonatal care of high quality to prevent these possible deaths. We recognise that the indicator measures only access and not quality of care.

Considering the sum of live births and fetal deaths as proxy of total pregnancies and considering those remaining at risk of present negative outcomes, such as fetal death and preterm live birth [12], the survival analysis carried out on the period 2000 and 2010 showed a reduction of 1604 fetal deaths, which corresponds to a 45 % of the total increase of preterm births over the period. This is therefore the maximum contribution to the increase in preterm births that could have resulted from effective interventions to prevent a fetal death leading to a preterm birth.

The present study was performed with secondary data files and we had limited number of confounding variables and recognize that others were not controlled and may be present. Although the data available does not enable the assessment of the etiological complexity of preterm births, the temporal analysis of these data suggests that the growth trend of preterm births is partly due to the increase of medically indicated interruption of pregnancy, aiming to reduce fetal losses. This may contribute to the increase idiopathic preterm births.

## Conclusions and recommendations

The improvement in prenatal and delivery care could be contributing to the reduction of fetal mortality by increasing survival of preterm pregnancies. Appropriate clinically indicated interventions in preterm pregnancies births may explain at least in part both the fall in fetal mortality and the increase in preterm live births, up to a maximum of 45 % of the additional in preterm births in the last 10 years.

Future studies of preterm births and fetal mortality should better elucidate the existing relationship between the reduction of fetal mortality and increase of preterm births.
